# Supplementary figures and images for: Minimum error correction-based haplotype assembly: Considerations for long read data
Source: PLoS One. 2020 Jun 12;15(6):e0234470. doi: 10.1371/journal.pone.0234470 (PMC7292361; doi:10.1371/journal.pone.0234470)

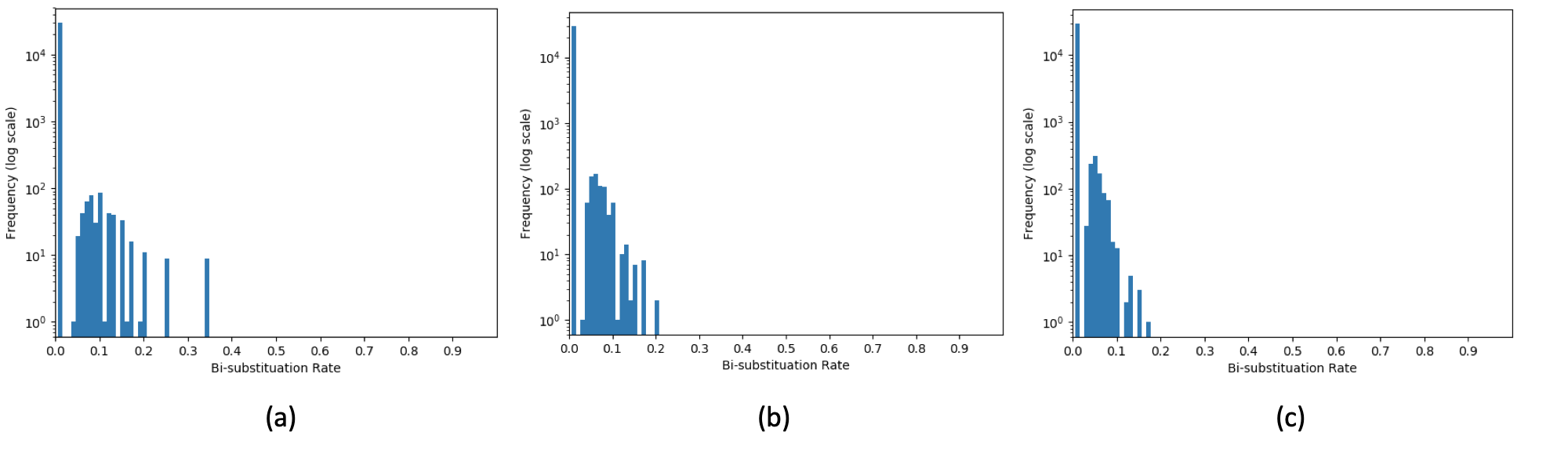

Supplement: S1 Fig — a: coverage 10. b: coverage 15. c: coverage 20. (TIFF) [file pone.0234470.s001.tiff]

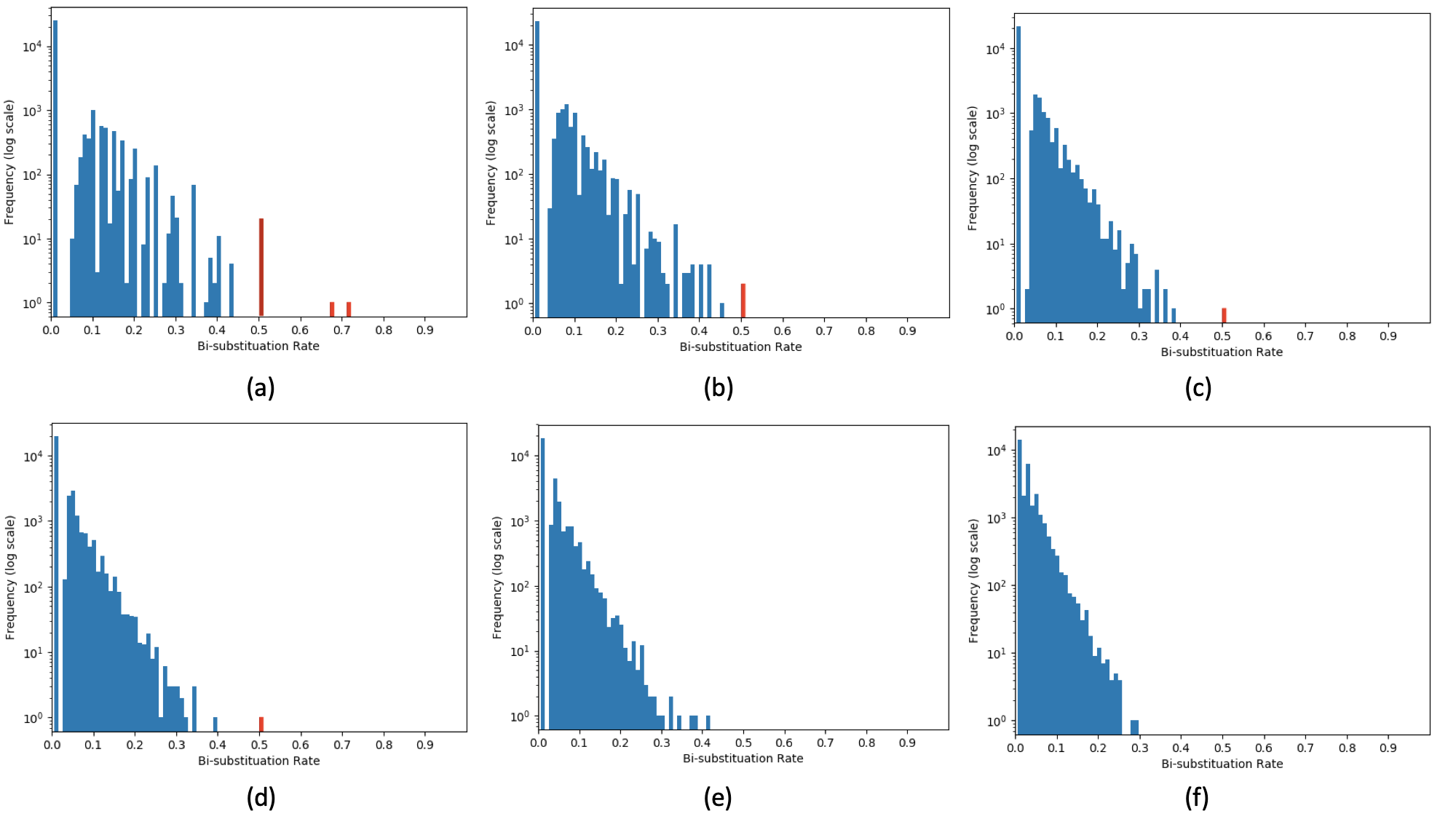

Supplement: S2 Fig — a: coverage 10. b: coverage 15. c: coverage 20. d: coverage 25. e: coverage 30. f: coverage 45. The red bars indicate results for which the antecedent of Theorem 1 is satisfied. (TIFF) [file pone.0234470.s002.tiff]
