# Supplementary material for: Minimum error correction-based haplotype assembly: Considerations for long read data
Source: PLoS One. 2020 Jun 12;15(6):e0234470. doi: 10.1371/journal.pone.0234470 (PMC7292361; doi:10.1371/journal.pone.0234470)
Supplement: S1 Appendix — A: Properties of extended hamming distance. B: Proof of Theorem 1. C: Properties of MEC. D: Extension to polyploid genomes. (ZIP) [file pone.0234470.s003.zip › S1_Appendix.pdf]

A Properties of extended hamming distance

The EHD function $D(\cdot,\cdot)$ is defined as

$$\mathrm{D}:\{0,1,-1\}^l\times\{0,1,-1\}^l\rightarrow\mathbb{R}^+\cup\{0\}, D(\boldsymbol{a},\boldsymbol{b})=\sum_{j=1}^{l} d(\boldsymbol{a}(j),\boldsymbol{b}(j)), \tag{A.1}$$

where

$$\mathrm{d}:\{0,1,-1\}\times\{0,1,-1\}\rightarrow\{0,1\},\quad d(a,b)=\begin{cases}1, \text{ if } a\neq 0 \text{ \& } b\neq 0 \text{ \& } a\neq b\\ 0, \text{ otherwise.}\end{cases} \tag{A.2}$$

This function is a distance if the following four conditions are satisfied:

$$\begin{array}{l}1.\ D(\boldsymbol{a},\boldsymbol{b})\geq 0\\ 2.\ \boldsymbol{a}=\boldsymbol{b}\Leftrightarrow D(\boldsymbol{a},\boldsymbol{b})=0\\ 3.\ D(\boldsymbol{a},\boldsymbol{b})=D(\boldsymbol{b},\boldsymbol{a}) \text{ (Symmtery)}\\ 4.\ D(\boldsymbol{a},\boldsymbol{c})\leq D(\boldsymbol{a},\boldsymbol{b})+D(\boldsymbol{b},\boldsymbol{c}) \text{ (Triangle inequality)}\end{array} \tag{A.3}$$

However, we show that this is not always the case and the EHD is an improper distance metric.

1. The first condition is always true due to the definition of EHD which is a summation over a series of $\{0,1\}$ and therefore is always nonnegative.
2. When $\boldsymbol{a}=\boldsymbol{b}$, then for all j, $d(\boldsymbol{a}(j),\boldsymbol{b}(j))=0$, which leads to the RHS result. However, the reverse is not always true. As an instance, for $\boldsymbol{a}=[010]$ and $\boldsymbol{b}=[-110]$, we have $D(\boldsymbol{a},\boldsymbol{b})=0$, while $\boldsymbol{a}$ and $\boldsymbol{b}$ are unequal. It is concluded that when $D(\boldsymbol{a},\boldsymbol{b})=0$, for the position of zero entries of $\boldsymbol{a}$, the corresponding entries of $\boldsymbol{b}$ can be either 1 or -1. Furthermore, this condition forces the corresponding nonzero entries of the two vectors to be equal. Therefore, this condition is true when all the entries are nonzero.
3. It is obvious that the EHD is symmetric due to the symmetry of $d(\cdot,\cdot)$.
4. The triangle inequality does not hold. A counter example is:

$$\boldsymbol{a}=[111],\boldsymbol{b}=[010],\boldsymbol{c}=[-101]\quad\Rightarrow\quad D(\boldsymbol{a},\boldsymbol{c})=1, D(\boldsymbol{a},\boldsymbol{b})=0, D(\boldsymbol{b},\boldsymbol{c})=0,$$

which yields to an unacceptable result $1\leq 0+0$. In fact, this condition holds when the locations of zero entries of $\boldsymbol{a}$, $\boldsymbol{b}$ and $\boldsymbol{c}$ are similar or all the entries are nonzero. Since EHD is part of the MEC function, the provided material in this section gives us an insight to understand the behaviour of MEC.

B Proof of Theorem 1

First, we discuss the fragment matrix model and then show in a lemma that changing the origin of each read does not affect the MEC function. Then a proof of Theorem 1 is given.

Fragment matrix model

As introduced in Methods Section, the fragment matrix model is given by

$$\boldsymbol{R}=P_\Omega(\boldsymbol{M})+\boldsymbol{E}, \tag{B.1}$$

where $\boldsymbol{E}$ is the error matrix discussed before and P_Ω is defined in (2). The completed matrix $\boldsymbol{M}$ is expressed as

$$\boldsymbol{M}=\boldsymbol{u}^T\boldsymbol{h}_{ex}. \tag{B.2}$$

Therefore the rank of matrix $\boldsymbol{M}$ is one. Each entry of the $1\times N$ origin vector $\boldsymbol{u}$ shows the haplotype from which each read originates that can be either $+1$ or -1 corresponding to the paternal or maternal haplotype, respectively.

Independency of MEC from origin of read

Lemma: Consider a given haplotype $\boldsymbol{h}_t$ and two fragment matrices $\boldsymbol{R}_a$ and $\boldsymbol{R}_b$ corresponding to two different origin vectors $\boldsymbol{u}_a$ and $\boldsymbol{u}_b$. We claim that if the error positions of the fragment matrices are the same, then both matrices are with equal MEC. This is presented in mathematical notation in (B.3).

$$\forall \boldsymbol{h}_t \quad \text{MEC}(\boldsymbol{R}_a, \boldsymbol{h}_t) = \text{MEC}(\boldsymbol{R}_b, \boldsymbol{h}_t), \tag{B.3}$$

where

$$\boldsymbol{R}_a = P_\Omega(\boldsymbol{u}_a^T \boldsymbol{h}) + \boldsymbol{E}_a, \tag{B.4}$$
$$\boldsymbol{R}_b = P_\Omega(\boldsymbol{u}_b^T \boldsymbol{h}) + \boldsymbol{E}_b, \tag{B.5}$$

in which $\boldsymbol{E}_a$ and $\boldsymbol{E}_b$ are the error matrices whose error positions are identical.

Proof. Since, each row of the fragment matrix affects the MEC independently, it is enough to prove the lemma only for the nth arbitrary row. To do so, we should prove that

$$\min\{D(\boldsymbol{r}_a, \boldsymbol{h}_t), D(\boldsymbol{r}_a, -\boldsymbol{h}_t)\} = \min\{D(\boldsymbol{r}_b, \boldsymbol{h}_t), D(\boldsymbol{r}_b, -\boldsymbol{h}_t)\}, \tag{B.6}$$

in which $\boldsymbol{r}_a$ and $\boldsymbol{r}_b$ are the nth rows of $\boldsymbol{R}_a$ and $\boldsymbol{R}_b$, respectively defined as

$$\boldsymbol{r}_a = P_{\Omega_n}(\boldsymbol{u}_a(n)\boldsymbol{h}) + \boldsymbol{e}_a, \tag{B.7}$$
$$\boldsymbol{r}_b = P_{\Omega_n}(\boldsymbol{u}_b(n)\boldsymbol{h}) + \boldsymbol{e}_b, \tag{B.8}$$

where $\boldsymbol{u}_a(n)$ and $\boldsymbol{u}_b(n)$ show the nth entries of $\boldsymbol{u}_a$ and $\boldsymbol{u}_b$ and $\boldsymbol{e}_a$ and $\boldsymbol{e}_b$ are the nth rows of $\boldsymbol{E}_a$ and $\boldsymbol{E}_b$, respectively. Also, $P_{\Omega_n}(\cdot)$ is a sub-operator of $P_\Omega(\cdot)$ dedicated to the nth row of a given matrix. Clearly, for $\boldsymbol{u}_a(n) = \boldsymbol{u}_b(n)$, we have $\boldsymbol{r}_a = \boldsymbol{r}_b$ and (B.6) is held. Otherwise, for $\boldsymbol{u}_a(n) \neq \boldsymbol{u}_b(n)$, without loss of generality, we assume that $\boldsymbol{u}_a(n) = 1$ and $\boldsymbol{u}_b(n) = -1$. Then, (B.8) and (B.9) reduce to

$$\boldsymbol{r}_a = P_{\Omega_n}(\boldsymbol{h}) + \boldsymbol{e}_a \tag{B.9}$$
$$\boldsymbol{r}_b = P_{\Omega_n}(-\boldsymbol{h}) + \boldsymbol{e}_b. \tag{B.10}$$

Considering that the error positions of $\boldsymbol{r}_a$ and $\boldsymbol{r}_b$ are identical and exploiting the model of (B.3), it can be shown that $\boldsymbol{e}_a = -\boldsymbol{e}_b$. Using this result in (B.10) and (B.11), we get $\boldsymbol{r}_a = -\boldsymbol{r}_b$. Using the first property of MEC (as shown in section C), we get

$$D(\boldsymbol{r}_a, -\boldsymbol{h}_t) = l_n - D(\boldsymbol{r}_a, \boldsymbol{h}_t) = l_n - D(\boldsymbol{r}_b, -\boldsymbol{h}_t) = D(\boldsymbol{r}_b, \boldsymbol{h}_t), \tag{B.11}$$

where l_n shows the number of nonzero entries of the nth row. By using (B.11) in the left side of (B.6), the lemma is proved as

$$\min\{D(\boldsymbol{r}_a, \boldsymbol{h}_t), D(\boldsymbol{r}_a, -\boldsymbol{h}_t)\} = \min\{D(\boldsymbol{r}_a, \boldsymbol{h}_t), l_n - D(\boldsymbol{r}_a, \boldsymbol{h}_t)\} = \min\{D(\boldsymbol{r}_b, -\boldsymbol{h}_t), D(\boldsymbol{r}_b, \boldsymbol{h}_t)\}. \tag{B.12}$$

This lemma which will be used in the next section shows that the MEC function is not sensitive to the changes of entries of $\boldsymbol{u}$ under the assumptions of the lemma.

Proof of Theorem 1

To prove Theorem 1, we propose a specific haplotype vector like $\boldsymbol{h}_d$ which leads to a lower MEC than the exact haplotype, meaning that minimizing the MEC function does not necessarily lead to the exact haplotype. To do so, first we suppose that the antecedent of Theorem 1 is held for the kth column, *i.e.*, $E^{(k)} > c^{(k)}/2$ and $\boldsymbol{h}_d$ is constructed as

$$\boldsymbol{h}_d = [\boldsymbol{h}_{ex}(1), \ldots, \boldsymbol{h}_{ex}(k-1), -\boldsymbol{h}_{ex}(k), \boldsymbol{h}_{ex}(k+1), \ldots, \boldsymbol{h}_{ex}(l)], \tag{B.13}$$

in which $\boldsymbol{h}_d(k)$ is equal to $-\boldsymbol{h}_{ex}(k)$. To prove that $\text{MEC}(\boldsymbol{R}, \boldsymbol{h}_d) < \text{MEC}(\boldsymbol{R}, \boldsymbol{h}_{ex})$, without loss of generality, based on provided lemma, we may consider $\boldsymbol{u} = [1, \ldots, 1]$. In such case, there is no difference to count

the mismatches for either the rows or columns of $\boldsymbol{R}$ due to the definition of MEC in (4). Therefore, we can write MEC as

$$\text{MEC}(\boldsymbol{R},\boldsymbol{h}) = \text{MEC}(\boldsymbol{R}^{(\sim k)},\boldsymbol{h}^{(\sim k)}) + \text{MEC}(\boldsymbol{r}^{(k)},\boldsymbol{h}(k)), \tag{B.14}$$

in which $\boldsymbol{r}^{(k)}$ is the kth column of $\boldsymbol{R}$ and $\boldsymbol{R}^{(\sim k)}$ shows a matrix whose k^{th} column has been omitted. Furthermore, based on the properties of the MEC function, as shown in section C, it can be seen that

$$\text{MEC}(\boldsymbol{r}^{(k)},\boldsymbol{h}(k)) = E^{(k)}, \tag{B.15}$$

$$\text{MEC}(\boldsymbol{r}^{(k)},-\boldsymbol{h}(k)) = c^{(k)} - E^{(k)}. \tag{B.16}$$

Therefore, using (B.15) and (B.16) in (B.14) for $\boldsymbol{h}_d$ and $\boldsymbol{h}_{ex}$, we get

$$\text{MEC}(\boldsymbol{R},\boldsymbol{h}_d) = \text{MEC}(\boldsymbol{R}^{(\sim k)},\boldsymbol{h}_{ex}) + c^{(k)} - E^{(k)}, \tag{B.17}$$

$$\text{MEC}(\boldsymbol{R},\boldsymbol{h}_{ex}) = \text{MEC}(\boldsymbol{R}^{(\sim k)},\boldsymbol{h}_{ex}) + E^{(k)}. \tag{B.18}$$

On the other hand, the antecedent of Theorem 1 results in $c^{(k)} - E^{(k)} < E^{(k)}$. Thus, (B.17) and (B.18) accomplish the proof.

C Properties of MEC

The MEC function is calculated for the fragment matrix $\boldsymbol{R}$ with the dimension of $N \times l$ and the haplotype $\boldsymbol{h}$ as:

$$\text{MEC} : \{0,1,-1\}^{N\times l} \times \{1,-1\}^{l} \to \mathbb{R}^{+} \cup \{0\}, \text{MEC}(\boldsymbol{R},\boldsymbol{h}) = \sum_{i=1}^{N} \min\{D(\boldsymbol{r}_i,\boldsymbol{h}), D(\boldsymbol{r}_i,-\boldsymbol{h})\}, \tag{C.1}$$

where $D(\cdot,\cdot)$ is defined by (4) and (5) and $\boldsymbol{r}_i$ shows the ith row of $\boldsymbol{R}$. The following properties can be shown for the MEC function.

1. For $\boldsymbol{r}_i$ with l_i known nonzero entries, by supposing $D(\boldsymbol{r}_i,\boldsymbol{h}) = D_i$, we get

$$\begin{aligned} D(\boldsymbol{r}_i,-\boldsymbol{h}) &= l_i - D_i,\\ D(-\boldsymbol{r}_i,\boldsymbol{h}) &= l_i - D_i,\\ D(-\boldsymbol{r}_i,-\boldsymbol{h}) &= D_i. \end{aligned} \tag{C.2}$$

2. For every $\boldsymbol{r}_i$ and $\boldsymbol{h}$, we have

$$D(\boldsymbol{r}_i,\boldsymbol{h}) = \begin{cases} k, & \text{if the } i^{th} \text{ read came from paternal haplotype,}\\ l_i - k, & \text{if the } i^{th} \text{ read came from maternal haplotype,} \end{cases} \tag{C.3}$$

where $k \in N$. For the exact haplotype $\boldsymbol{h}_{ex}$, k is equal to the number of error entries of $\boldsymbol{r}_i$ denoted by e_i. Then, the MEC for the exact haplotype is

$$\text{MEC}(\boldsymbol{R},\boldsymbol{h}_{ex}) = \sum_{i=1}^{N} \min\{e_i, l_i - e_i\} = E, \tag{C.4}$$

in which E is the total number of error in the fragment matrix. For the error-free fragment matrix and the paternal exact haplotype $\boldsymbol{h}_{ex}$, (C.4) reduces to $\text{MEC}(\boldsymbol{R},\boldsymbol{h}_{ex}) = 0$.

D Extension to polyploid genomes

Some animals and plants are polyploids. They contain more than two copies of each chromosome. In such case, our modeling presented in (3) and (B.2) may be generalized to

$$\boldsymbol{M} = \boldsymbol{U}^T \boldsymbol{H}_{ex}, \tag{D.1}$$

in which $\boldsymbol{H}_{ex}$ contains P haplotypes and $\boldsymbol{U}$ shows the haplotypic origin of each read. The definition of MEC for fragment matrix $\boldsymbol{R}$ and candidate haplotype $\boldsymbol{H}_c$ can be generalized to

$$\text{MEC}(\boldsymbol{R}, \boldsymbol{H}_c) = \sum_{i=1}^{N} \min_{p} D(\boldsymbol{r}_i, \boldsymbol{H}_c\{p\}), \tag{D.2}$$

in which $\boldsymbol{H}_c\{p\}$ is the pth haplotype (i.e. p^{th} row of $\boldsymbol{H}_c$).

Here, we consider Theorem 1 (presented in the Methods section) for the polyploid case to show that MEC failure can happen in a specific polyploid case as well. Suppose that the number of reads originating from the pth haplotype covering the tth SNP is $c^{(t)}\{p\}$ and the number of erroneous entries in the tth SNP is $E^{(t)}\{p\} > c^{(t)}\{p\}/2$.

We define a haplotype matrix $\boldsymbol{H}_d$ and then show that its MEC is lower than that of the exact haplotype $\boldsymbol{H}_{ex}$. These two matrices are the same except the $(p, t)^{th}$ element is such that $\boldsymbol{H}_d(p, t) = -\boldsymbol{H}_{ex}(p, t)$. In a simplified case, suppose that P haplotypes are separated enough such that the change in one element does not affect the estimation of haplotypic origin of the reads (the value of $\text{argmin}_p D(\boldsymbol{r}_i, \boldsymbol{H}_c\{p\})$. Thus, due to the definition of MEC in (E.2), we can write

$$\text{MEC}(\boldsymbol{R}, \boldsymbol{H}_d) = \text{MEC}(\boldsymbol{R}\{\sim p\}, \boldsymbol{H}_d\{\sim p\}) + \sum_{i} D(\boldsymbol{r}_i, \boldsymbol{H}_d\{p\}), \tag{D.3}$$

in which $\boldsymbol{R}\{\sim p\}$ is the submatrix of $\boldsymbol{R}$ restricted to those rows originating from all P haplotypes except p^{th}. Last term is a summation over those rows originating from p^{th} haplotype. Due to the definition of $\boldsymbol{H}_d$ and $\boldsymbol{H}_{ex}$, we have

$$\text{MEC}(\boldsymbol{R}\{\sim p\}, \boldsymbol{H}_d\{\sim p\}) = \text{MEC}(\boldsymbol{R}\{\sim p\}, \boldsymbol{H}_{ex}\{\sim p\}). \tag{D.4}$$

Due to the assumption on erroneous entries, we have $\sum_i D(\boldsymbol{r}_i, \boldsymbol{H}_d\{p\}) < \sum_i D(\boldsymbol{r}_i, \boldsymbol{H}_{ex}\{p\})$ which results in

$$\text{MEC}(\boldsymbol{R}, \boldsymbol{H}_d) < \text{MEC}(\boldsymbol{R}, \boldsymbol{H}_{ex}). \tag{D.5}$$

This shows that optimizing the MEC function does not guarantee reaching the exact (true) haplotypes.
